# Pancreatic solid pseudopapillary neoplasm in male patients: systematic review with three new cases

**DOI:** 10.1007/s13304-020-00905-4

**Published:** 2020-10-29

**Authors:** Anna Caterina Milanetto, Anna-Lea Gais Zürcher, Lorenzo Macchi, Alina David, Claudio Pasquali

**Affiliations:** Clinica Chirurgica 1, Pancreatic and Endocrine Digestive Surgical Unit, Department of Surgery, Oncology and Gastroenterology, Università degli Studi di Padova, via Giustiniani, 2 - 35128 Padua, Italy

**Keywords:** Solid pseudopapillary neoplasm, Male patient, Pancreatic tumour, Pancreatic surgery

## Abstract

Pancreatic solid pseudopapillary neoplasm (pSPN) is a rare exocrine neoplasm, which generally occurs in young women. This study analyses the clinical characteristics of pSPN in male patients through a systematic review of the literature, adding three new cases from our institution. We reviewed our experience in Pspns, and we performed a systematic review of pSPN of all articles published in English in PubMed and SCOPUS from 1980. Using the final included articles, we evaluated clinic-pathological features, surgical treatment and prognosis of male patients affected by pSPN. From the literature review and our cases, we collected 246 male patients with a proven pSPN. Mean age was 34.3 (range 4–78) years, with 26.2% patients younger than 18 years. Patients were asymptomatic in 35.9% of cases, despite a mean tumour size of 6.3 cm. In 63.7% of cases, the pSPN was located in the body–tail region. Distant metastases were reported at diagnosis in only 10 (4.1%) patients. A correct pre-operative diagnosis (including cytopathology) was provided in 53.6% of patients, with only 40 fine-needle aspiration/biopsy performed. Standard pancreatic resections represented 90.4% of surgical procedures. Beta-catenin and progesterone receptors were positive at immunostaining in 100% and 77.8% of cases, respectively. Fourteen (7.2%) patients relapsed after a mean disease-free survival of 43.1 months. After a mean follow-up of 47 (range 4–180) months, 89.5% of patients were alive and disease-free. Although rare, when dealing with a solid-cystic pancreatic mass, even in asymptomatic male patients, a pSPN should be considered as a possible diagnosis.

**Electronic supplementary material:**

The online version of this article (10.1007/s13304-020-00905-4) contains supplementary material, which is available to authorized users.

## Introduction

In 1959, the pathologist Virginia Kneeland Frantz firstly described a solid pseudopapillary neoplasm (SPN) of the pancreas in a 2-year-old male patient [1]. It is a low-grade malignant tumour lacking a specific line of pancreatic epithelial differentiation [2], accounting for 1–3% of all pancreatic tumours [3]. Solid pseudopapillary neoplasm is known for its strong female preponderance (F:M = 10:1); in fact, it affects women less than 30 years old in 85% of cases [4], while men are rarely affected. About 20–25% of SPNs occur in patients younger than 18 years [4].

The clinical presentation is usually non-specific. The most frequent symptoms are abdominal pain or discomfort, a palpable mass, and compression of the stomach, duodenum, or main biliary duct related to the large size of the tumour [5]. Some patients are completely asymptomatic, and the SPN may be detected incidentally by imaging studies, or by routine physical examination. Laboratory tests are normal, hormonal activity is absent and tumour markers are generally unremarkable [5].

A complete surgical excision is curative in patients with a SPN limited to the pancreas. Up to 10–15% of SPNs show malignant behaviour and distant metastases, usually to the liver and peritoneum [6]. Nevertheless, SPN is generally associated with an excellent long-term prognosis, with a reported 10-year disease-specific survival rate of 96% [7], even when including the resection of distant metastases [8]. Currently, no specific chemotherapy regimens are available.

In recent years, the reported number of SPNs in the English literature has increased sevenfold since 2000 [9]. The knowledge of this rare pancreatic tumour has become better than before, and the quality and use of cross-sectional imaging has improved [9]. The interest in this disease is increasing, especially in those rare SPNs that develop in male patients. In this specific field, the available literature is limited. We report three new cases of pancreatic SPN in male patients, and we performed a review of the English literature of pancreatic SPN in males from 1980, evaluating their clinic-pathological features, surgical treatment and outcome.

## Patients and methods

### Literature search

The PRISMA (Preferred Reporting Items for Systematic Reviews and Meta-Analyses) guidelines were followed when performing and reporting this systematic review [10]. PubMed and SCOPUS were queried from January 1, 1980 to May 12, 2020, using predetermined search strings (Appendix 1). Because of the various definitions associated with SPN of the pancreas, according to the WHO classification of tumours 2019 [2], the related terminology was used in the search strategy, that included the terms “pancreas”, “pseudopapillary”, “solid cystic” and “papillary cystic” tumour.

### Inclusion criteria

Full-text studies published in English language after 1980 were included. In 1981, the use of computed tomography (CT) scan was first reported for SPN diagnosis [11], increasing the diagnostic accuracy for pre-operative diagnosis, and in 1981 Klöppel et al. [12] described the SPN as a well-defined pancreatic cystic tumour. After deduplication of common reports between PubMed and SCOPUS, all publications related to pancreatic SPNs (histologically or cytologically confirmed), which included male patients and reported a description of patient and tumour characteristics (demographics, diagnosis, treatment, and outcome) were considered for the eligibility phase.

### Studies selection and data extraction

Two investigators (A.G.Z. and A.D.) independently reviewed all the records left after the screening phase. In case of disagreement, a third investigator (A.C.M.) resolved the conflict. We excluded full texts not found from the available resources. Case series including female patients with aggregate clinical data were excluded. To avoid duplication of cases, the clinical data reported were cross-referenced by the country of origin, and then by the centre from which the case originated. Variables that were recorded included: patient age and symptoms; tumour features (size, location, and presence of distant metastases); pre-operative diagnosis (imaging studies, fine-needle aspiration-FNA or biopsy); type of surgery; immunohistochemical data (β-catenin, alpha1-antitrypsin, vimentin, progesterone receptor, CD10, and CD56); time of follow-up; complementary treatment performed at diagnosis or after disease recurrence (i.e. chemotherapy/systemic treatment, loco-regional treatment); and final outcome (disease recurrence, disease-free survival and status). If some data of the selected studies were reported as aggregate with that of female patients, these were considered as “not available”. When data were reported for some but not all patients, we presumed that the finding was present in the patients reported and absent in the others.

### Institution data search and case presentation

Male patients who underwent surgery for a pancreatic SPN from January 1986 to December 2017 in the study centre were enrolled. Their clinical records were retrieved, and retrospectively evaluated from clinical charts. The same data collected from the literature search were analysed for the cases from our institution, and in addition, we reported pancreatic tumour markers, and mitotic index and/or Ki-67 labelling index. All the patients had a regular follow-up, with clinical evaluation and imaging studies (CT scan, and/or magnetic resonance imaging-MRI). Follow-up closed in December 2019. The status of patients was defined at the last follow-up visit by medical reports.

## Results

### Literature selection and systematic review

**Fig. 1:**
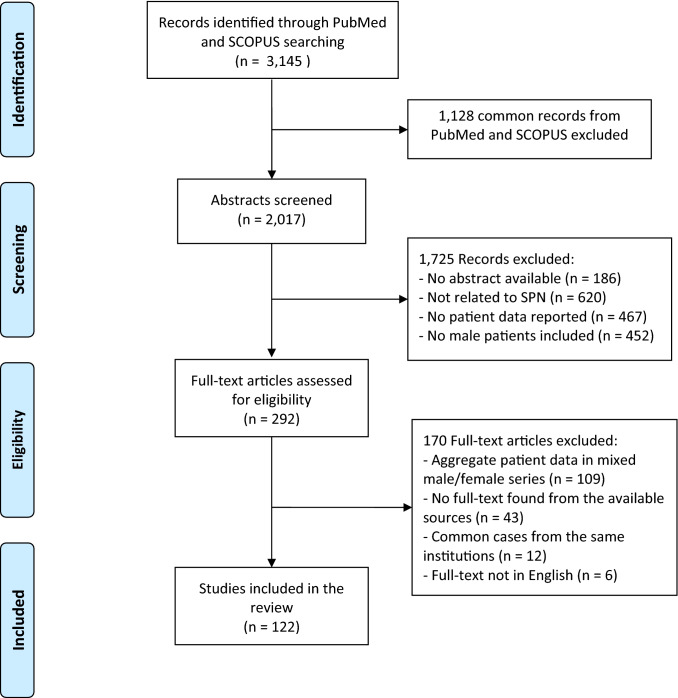
Flow diagram illustrating the search process

The literature search generated 3145 reports, and after deduplication and screening, 292 full-text articles met the inclusion criteria (Fig. 1). Twelve studies were excluded because they reported on common cases from the same institutions. Six articles with English full text not available and 43 full texts not found from the available sources were also excluded. Other 109 articles reporting aggregate male and female patients’ data were not eligible for the review. Finally, 122 studies were included in the systematic review for qualitative synthesis (Appendix 2). These included 49 (40.2%) case reports and 73 (59.8%) case series of female and male patients. Most studies (107) were published after 2000, with only 15 studies published between 1980 and 2000. We reviewed 243 cases of pancreatic SPN in male patients.

### Presentation of our cases

From 1986 to 2017, 19 patients with pancreatic SPNs were operated in our Surgical Unit. Three of them (15.8%) were male patients (Table 1).

**Table 1:** Clinic-pathological features, surgical treatment and outcome of patients from our institution (*n* = 3)

	Case 1	Case 2	Case 3
Age (years) / Observation year	75 / 2010	20 / 2017	14 /2017
Symptoms	No	Abdominal discomfort, weight loss	Previous mild pancreatitis, fever
Tumour Site / Size (cm)	Tail / 4.5	Tail / 5.7	Head / 2.9
Imaging studies	US, CT scan, MRI, ^18^F-FDG PET-CT	CT scan, MRI, ^18^F-FDG PET-MRI	US, MRI, EUS-FNB, ^18^F-FDG PET-MRI
18F-FDG PET SUVmax	negative	6.5	4.8
Pre-operative diagnosis / Pre-operative biopsy	SCA / no	SPN / no	SPN / β-catenin+
Pancreatic tumour markers	CA19.9 negative	CEA, CA19.9, α-FP, CgA, NSE negative	CEA, CA19.9, α-FP, CgA, NSE negative
Surgery	DP	DP	PD
Lymph node and/or distant metastases	no	no	no
Mitotic Index and/or Ki-67	0/10 HPF	n.a	0–1/10 HPF – 3%
IHC	β-catenin+ PGR+ NSE+ CD10+ α1-AT+	β-catenin+ PGR+ NSE+ Cg- CEA -	PGR+ α1-AT+ NSE+ α1-ACT+
Follow-up (months)	115	34	25
Disease recurrence / Status	No / ANED	No / ANED	No / ANED

*SPN* solid pseudopapillary neoplasm, *US* abdominal ultrasound, *CT scan* computed tomography, *MRI* magnetic resonance imaging, ^*18*^*F-FDG PET* ^18^F- fluorodeoxyglucose positron emission tomography, *EUS* endoscopic ultrasound, *FNB* fine-needle biopsy, *SUV max* standardized uptake value maximum, *SCA* serous cystadenoma, *β-catenin* Beta-catenin, *Ca19.9* carbohydrate antigen 19.9. CEA, carcinoembryonic antigen, *α-FP* alpha-feto protein, *CgA* chromogranin A, *NSE* neuron-specific enolase, *DP* distal pancreatectomy, *PD* pancreaticoduodenectomy, *HPF* high power fields, *n.a.* not available, *IHC* immunohistochemistry, *PGR* progesterone receptors, *α1-AT* alpha1-antitrypsin, *Cg* chromogranin staining, *α1-ACT* alpha1-antichymotrypsin, *ANED* alive and no evidence of disease

#### Case 1

**Fig. 2:**
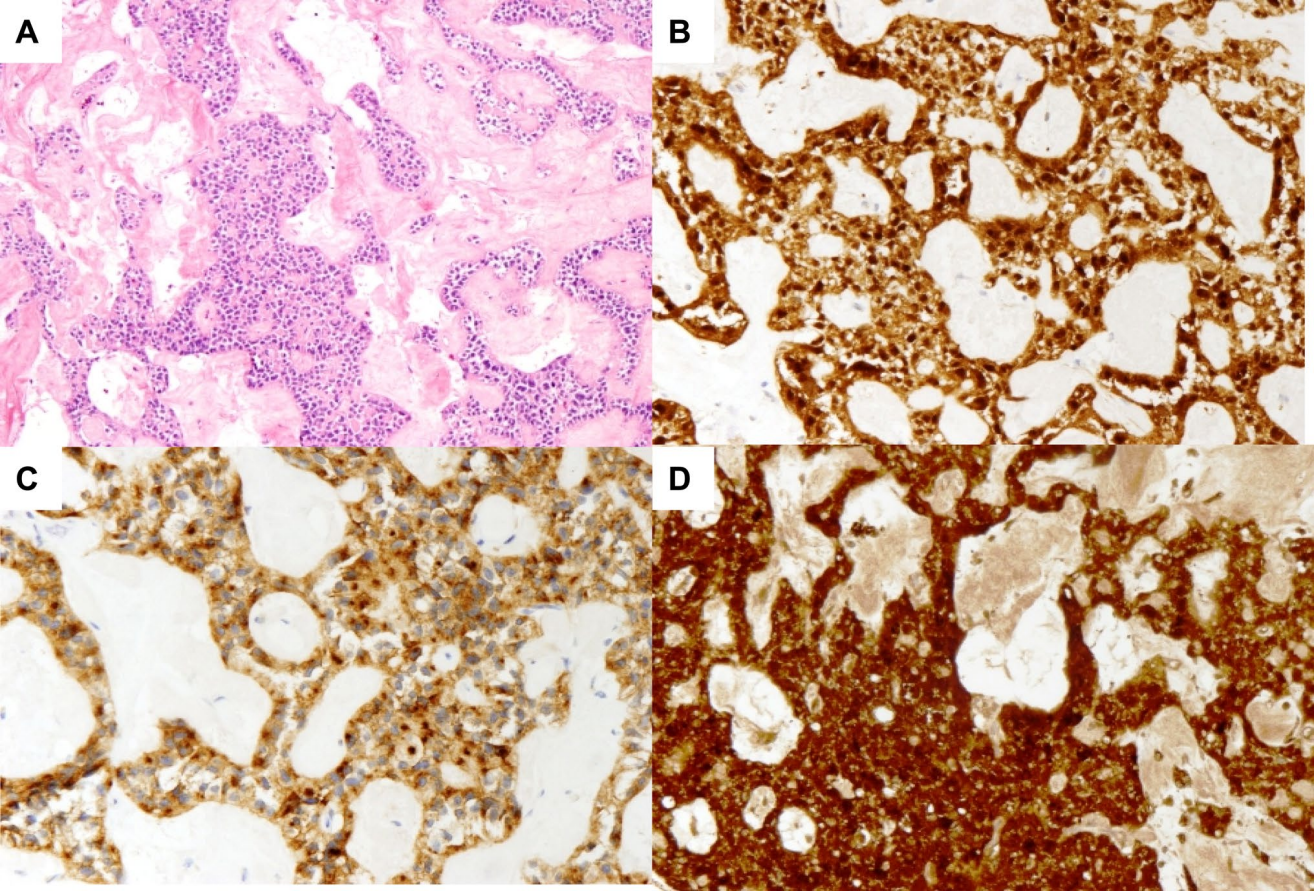
Representative H&E stain (**1a**), β-catenin immunostaining (**1b**), CD-10 immunostaining (**1c**), α-1 anti-trypsin and neuron-specific enolase immunostaining (**1d**) of case n.1

In 2010, an asymptomatic 75-year-old man was incidentally diagnosed at the abdominal ultrasound (US) with a solid lesion close to the splenic hilum. The CT scan confirmed a 4.5 cm, hypodense, solid mass with a minor cystic component, localised at the tail of the pancreas. A partially cystic signal intensity with peripheral dishomogeneous enhancement and multiple septa within the cystic component was evident at MRI. The ^18^F- fluorodeoxyglucose (FDG) positron emission tomography (PET)-CT showed no abnormal tracer uptake, and CA19.9 was within the normal range. The radiologist suspected a large serous cystadenoma, but due to the uncommon prevalent solid component, the surgeon considered safer to resect it and the patient underwent distal pancreatectomy. Pathology showed a pancreatic SPN, with a central haemorrhagic area. Mitotic index was 0/10 HPF, and immunohistochemistry was positive for β-catenin (Fig. 2) and progesterone receptors. The post-operative course was uneventful. The patient was alive without disease 115 months after surgery.

#### Case 2

In 2017, a 20-year-old man presented with upper abdominal discomfort and weight loss since 1 year. A CT scan revealed a dishomogeneous mass of 5.7 cm in the pancreatic tail, with a fluid appearance with a dense component, and a complete encasement of the splenic vein. The MRI confirmed a 5.0 cm mass with a dishomogeneous fluid content, and a peripheral anterior solid component. At the ^18^F-FDG PET-MRI, the pancreatic mass showed a hypermetabolic border (SUVmax 6.5) and a hypometabolic core. Tumour markers were negative. The patient underwent distal pancreatectomy for suspected SPN. Histology reported a SPN with large necrotic areas, infiltration of the pseudocapsule and perineural invasion. Immunohistochemistry was positive for β-catenin, progesterone receptors and NSE. The patient had an uneventful post-operative course. He had no evidence of disease recurrence 34 months after surgery.

#### Case 3

**Fig. 3:**
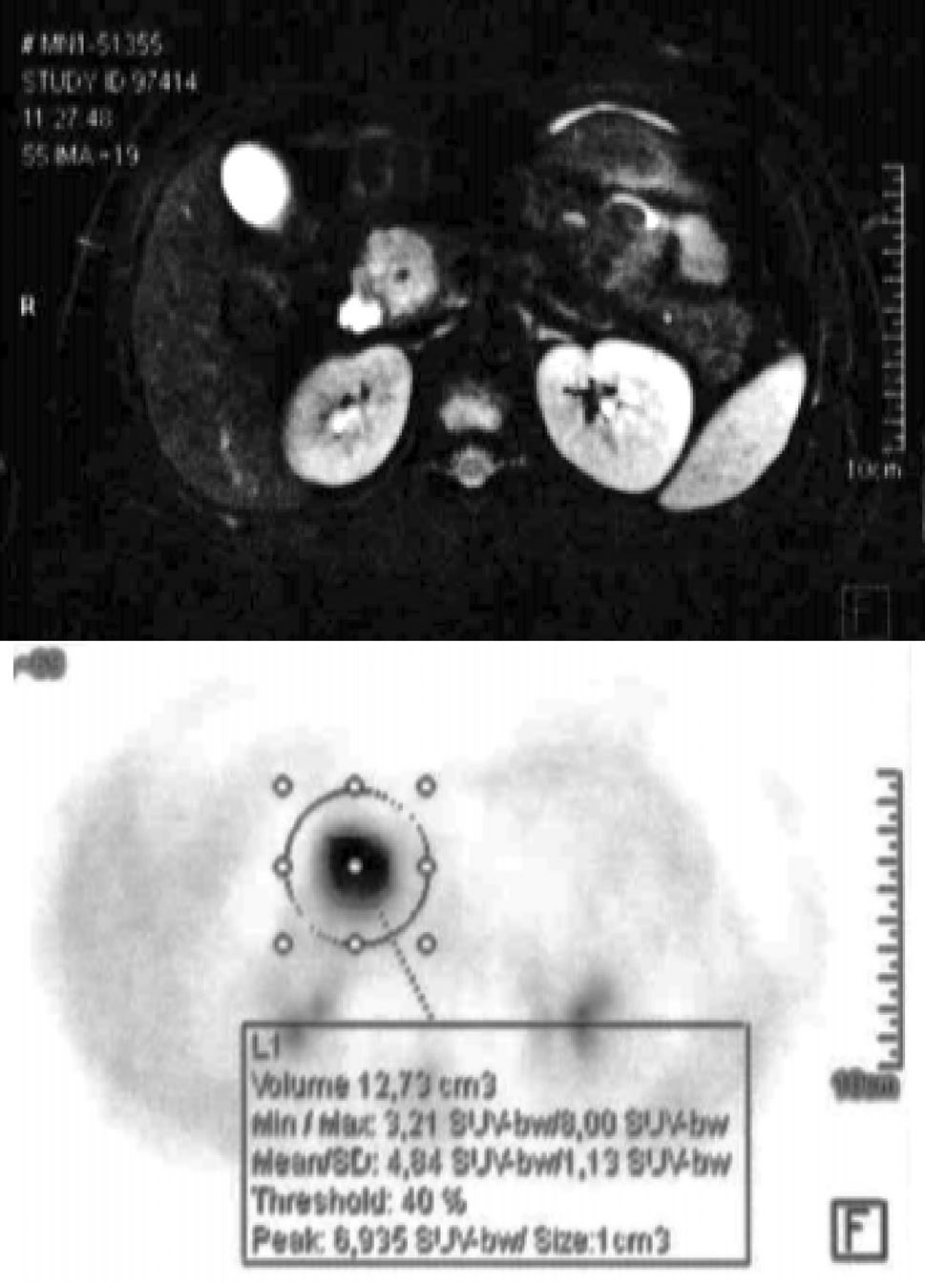
^18^F-FDG PET-MRI of case n.3: 2.9 cm in size SPN of the pancreatic head, with an intense tracer uptake (SUVmax 4.8)

In 2017, a 14-year-old boy underwent abdominal US, which revealed a 3 cm in size mass of the pancreatic head with hyperechoic spots. He had an episode of acute pancreatitis in the history, and since 1 year, he complained of a fever of unknown origin. The ^18^F-FDG PET-MRI confirmed the presence of a 2.9 cm mass in the pancreatic head, with an intense tracer uptake (SUVmax 4.8) (Fig. 3). Serum tumour markers were unremarkable. An Endoscopic US (EUS) with FNA-biopsy was also performed, and histology confirmed the suspect of SPN with a positive immunostaining for β-catenin. He underwent pancreatico-duodenectomy. Histology of the specimen showed a SPN without perivascular/perineural invasion. Mitotic index was 0–1/10 HPF, and immunostaining was positive for progesterone receptors, alpha-1-antichymotrypsin, alpha-1-antitrypsin and neuron-specific enolase. The patient developed a pancreatic biochemical leak in the post-operative course. He was alive without disease 25 months after surgery.

### Clinical features and preoperative diagnosis

The three cases from our institution were included in the systematic review. Clinic-pathological features, surgical treatment and outcome are summarised in Table 2. There were 246 male patients with a pancreatic SPN, and mean age at presentation was 34.3 (range 4–78) years. Only 58 (26.2%) patients were younger than 18 years old. They mostly presented with abdominal pain or discomfort, but more than one-third (35.9%) were asymptomatic. The SPN was located in the body–tail region of the pancreas in 63.7% of cases, and mean tumour size was 6.3 (range 0.5–26) cm. Ten (4.1%) patients presented with distant (mostly liver) metastases. In 12 (5.5%) patients, pre-operative imaging studies included ^18^F-FDG PET-CT, and SPNs showed an intense tracer uptake in 80% of cases. In 40 (19.1%) patients, FNA was performed, resulting in a correct identification of SPNs in 82.5% of cases. When considered together, pre-operative imaging studies and cytopathological examination allowed a correct pre-operative diagnosis in 53.6% of patients, whereas other pancreatic neoplasms (i.e. ductal or acinar adenocarcinoma, neuroendocrine neoplasm-NEN, unspecified pancreatic neoplasm), or a pseudocyst represented the alternative diagnoses leading the patients to surgery.

**Table 2:** Clinic-pathological features, surgical treatment and outcome (*n* = 246)

Male patients with SPN, *n*	246
Total no. patients in studies reporting age	221
Age, mean (range), years	34.3 (4–78)
Total no. patients in studies reporting symptoms	170
Symptoms, *n* (%)	
Yes	109 (64.1)
No	61 (35.9)
Total no. patients in studies reporting tumour size	217
Tumour size, mean (range), cm	6.3 (0.5–26.0)
Total no. patients in studies reporting tumour location	245
Tumour location, *n* (%)	
Head	81 (33.0)
Neck	8 (3.3)
Body-tail	156 (63.7)
Total no. patients in studies reporting distant metastases	244
Distant metastases at diagnosis, *n* (%)	
Yes	10 (4.1)
No	234 (95.9)
Total no. patients in studies reporting pre-operative imaging	218
^18^F-FDG PET/CT performed, *n* (%)	12 (5.5)
Available results	10
Intense tracer uptake	8 (80.0)
SUVmax, mean (range)	5.0 (3.4–6.5)
Total no. patients in studies reporting pre-operative FNA/biopsy	209
Pre-operative FNA/biopsy, *n* (%)	
Performed	40 (19.1)
Not performed	169 (80.9)
Correct diagnosis of SPN	33 / 40 (82.5)
Total no. patients in studies reporting pre-operative diagnosis	84
Correct diagnosis of SPN	45 (53.6)
Other pancreatic neoplasm/pseudocyst	39 (46.4)
Total no. patients in studies reporting surgical treatment	212
Surgery, *n* (%)	
Yes	206 (97.2)
Palliative/No	6 (2.8)
Total no. patients in studies reporting type of surgery	197
Type of surgery, *n* (%)	
PD	61 (31.0)
DP/SPDP	113 (57.4)
Total pancreatectomy	4 (2.0)
Other (CP, DPPHR, enucleation)	19 (9.6)
Total no. studies reporting IHC	76
IHC (positive/total), *n* (%)	
β-catenin	39 / 39 (100)
Alpha-1-antitrypsin	36 / 37 (97.3)
Vimentin	47 / 51 (92.2)
Progesterone receptors	28 /36 (77.8)
CD10	34 / 36 (94.4)
CD56	28 / 28 (100)
Total no. patients in studies reporting follow-up time	141
Follow-up, mean (range), months	47.0 (4–180)
Total no. patients in studies reporting other treatment than surgery	183
Performed	11 (6.0)
At diagnosis	5 (45.5)
After disease recurrence	6 (54.5)
Chemotherapy/systemic treatment	10 (90.9)
Loco-regional treatment/Re-do surgery	4 (36.4)
Total no. patients in studies reporting disease recurrence	195
Disease recurrence, *n* (%)	
Yes	14 (7.2)
No	181 (92.8)
Total no. patients in studies reporting DFS in disease recurrence	11
DFS, mean (range), months	43.1 (6–96)
Total no. patients in studies reporting patient status	200
Status, *n* (%)	
ANED	179 (89.5)
AWD	12 (6.0)
DOD	8 (4.0)
DOC	1 (0.5)

*SPN* solid pseudopapillary neoplasm, ^*18*^*F-FDG PET-CT* ^18^F- fluorodeoxyglucose positron emission tomography-computed tomography, *SUV* standardized uptake value, *FNA* fine-needle aspiration, *PD* pancreatico-duodenectomy, *DP* distal pancreatectomy, *SPDP* spleen-preserving distal pancreatectomy, *CP* central pancreatectomy, *DPPHR* duodenum-preserving pancreatic head resection, *IHC* immunohistochemistry, *β-catenin* beta-catenin, *DFS* disease-free survival, *ANED* alive and no evidence of disease, *AWD* alive with disease, *DOD* died of disease, *DOC* died of other cause

### Surgical treatment and pathological features

The vast majority (97.2%) of patients underwent pancreatic resection. Surgery consisted mostly in standard pancreatic resections (pancreatico-duodenectomy, distal pancreatectomy, and total pancreatectomy) performed in 90.4% of patients, and in 19 (9.6%) limited pancreatic resections (central pancreatectomy, duodenum-preserving pancreatic head resection, and enucleation). Among 10 patients presenting with distant metastases, 2 patients had adjuvant chemotherapy after surgery, 2 patients underwent surgical resection only, and 3 patients received only chemotherapy (i.e. gemcitabine, 5-fluorouracil) and/or trans-arterial embolization (TAE) of liver metastases; four of them died of disease after a mean follow-up of 6.7 months. Seventy-six studies reported on immunohistochemistry, excluding case series with aggregate male/female data. Beta-catenin resulted positive in all cases available, as well as CD56, while progesterone receptors were positive in 77.8% of cases.

### Follow-up and outcome

Follow-up time data were available for 141 (57.3%) patients, and mean follow-up was 47.0 (range 4–180) months. Fourteen (7.2%) patients showed a disease recurrence, after a mean disease-free survival (DFS) of 43.1 (range 6–96) months after standard pancreatic resections. Six of them received a treatment, which consisted of various chemotherapy regimens (i.e. gemcitabine, 5-fluorouracil, capecitabine, docetaxel, oxaliplatin, cisplatin, and irinotecan), in four patients associated with loco-regional treatments (i.e. TAE, microwave ablation, radiotherapy) or re-do surgery. Five of these patients were alive with disease after initial surgery (range 55–180 months). Patient outcome was reported in 200 (81.3%) cases, and most (89.5%) patients were alive without disease.

## Discussion

Solid pseudopapillary neoplasm (SPN) of the pancreas is a rare neoplasm. It mainly affects young women, suggesting a role form hormonal factors, but no association with endocrine diseases has been reported so far [2]. Its natural history is still uncertain, especially in those rare SPNs that develop in male patients. This neoplasm may derive from genital ridge-related cells [13], or pluripotent stem cells of the genital ridges [14] that become attached to the primordial pancreas during embryogenesis. Moreover, extra-pancreatic SPNs have been reported in retropancreatic tissue, ovary, and testis [2]. A systematic review on SPNs by Law et al. [9] reported of 336 SPNs in men collected up to 2012 (12% of the whole cases), as from the experience of our institution, with 15.8% of males among all patients operated for a SPN in the last 30 years. The present systematic review comprised 1052 total SPNs, with a SPN rate in males of 23.4%, resulting higher than previously reported. However, this finding is due to the article selection process, which provided for the exclusion of series including female patients only.

In male patients, SPNs show an older age at presentation when compared with that in female patients [6, 15, 16]. In the present review, the oldest patient was 78 years old, and adult patients represented 73.8% of total SPNs in males (mean age, 42.2 years). The late occurrence of SPN in males might be the result of a long-term exposure of the ectopic ovarian like stroma to normal level of female sex hormones [16], since normal levels of both progesterone and estrogen were detected in male patients [16]. When comparing young (less than 18 years old) and adult male patients with SPNs, the former showed a slightly larger mean tumour size than the latter (7.0 cm vs. 5.7 cm), as Bender et al. reported of a mean SPN size of 8.2 cm in young patients [17]. Regarding tumour site, adult male patients showed a SPN located in the body–tail region of the pancreas in the majority (65.8%) of cases, whereas in young males, SPNs may be located in the pancreatic head and body–tail in 41.4% and 56.9% of cases, respectively. This finding may suggest that distal pancreatic lesions, which are less likely symptomatic, may be detected only later in life, irrespective of their time of onset.

The clinical presentation of SPN usually consists of non-specific symptoms (i.e. abdominal pain or abdominal discomfort), and patients may be even asymptomatic. The number of incidentally detected SPNs has grown, accounting for about 40% of all SPN cases (irrespective of gender) [9], and in our systematic review 35.9% of patients were asymptomatic at diagnosis. No tumour markers or routine laboratory parameters have been identified for the diagnosis of SPNs, thus pre-operative diagnosis is based on imaging studies and cytopathological examination of FNA samples. Pre-operative diagnosis of SPNs may be challenging, especially in male patients for whom this type of exocrine neoplasm is less often suspected. In these patients, the main alternative diagnosis may be a pancreatic NEN. The introduction of CT scan in the 1980s has increased the pre-operative diagnostic accuracy for SPNs. Ultrasound, CT scan and MRI typically show a large well-circumscribed, heterogeneous mass with varying solid and cystic components [18], demarcated by peripheral contrast enhancement corresponding to a fibrous pseudocapsule, and occasional calcifications. Recently, Wang et al. [19] reported that calcifications and enhanced solid components within the unenhanced cystic components (defined “floating cloud” sign) are useful features in discriminating SPNs from hypodense pancreatic NENs at CT scan [19].

In our systematic review, most of the patients underwent a CT scan, but an ^18^F-FDG PET-CT (or PET-MRI) was performed in only 12 (5.5%) patients. Although the intensity of ^18^F-FDG uptake in SPNs may vary widely due to tumour heterogeneity [20], in a previous study [21] we showed that only two (3%) out of 69 SPNs collected in the literature were PET negative. Some authors compared the CT [22–24] and MRI [25] imaging features of pancreatic SPNs between male and female patients. Males were significantly older than female patients, and irrespective of tumour size, SPN in males had mainly a solid component, whereas female patients had a significantly high rate of cystic lesions. The high metabolism of SPNs at FDG-PET seems to have a direct relationship with tumour cellularity [26] and with the solid (cellular) component of SPNs [26], irrespective to malignant behaviour. Thus, SPNs with a predominant solid component, as those detected in males, can be easily identified based on a marked avidity for ^18^F-FDG (SUVmax range 3.5–18.3) [27], and differentiated from pancreatic NENs, which usually have a poor ^18^F-FDG uptake and a low SUVmax value [27]. In the present review, SPNs showed an intense tracer uptake at FDG-PET in 80% of cases (mean SUVmax 5.0). In the presence of a pancreatic mass suspected for a SPN or a NEN, even in case of liver metastases, surgery would be the treatment of choice. However, a correct diagnosis may be crucial in patients not fit for surgery, giving them the chance of a correct alternative treatment (i.e. somatostatin analogues for NENs).

Some authors [28] stated that radiologic diagnosis is sufficient for SPNs, especially when planning surgery. However, obtaining a pre-operative histologic diagnosis may be sometimes advisable, and EUS-guided FNA is the most frequently used procedure and a valuable technique for its diagnostic accuracy [29–31]. In the present review, when considered together imaging studies and FNA allowed a correct pre-operative diagnosis in 53.6% of cases. Notably, only 40 (19.1%) patients underwent a pre-operative FNA, which resulted positive for SPN in 82.5% of cases. Since a low amount of material may be available through FNA and immunohistochemistry is mandatory for diagnosis, the correct diagnostic antibody panel should be accurately chosen [32]. In young patients, FNA may not differentiate between SPN and pancreatoblastoma [33], whereas in adults a misdiagnosis with a pancreatic NEN may be avoided detecting the highly specific patterns of E-cadherin and β-catenin staining [34].

Histologically, SPNs are characterised by solid areas alternated with a pseudopapillary pattern and cystic spaces. These features result from degenerative changes occurring in the solid neoplasm [35], without increased mitoses or cytological atypia [36, 37]. These neoplasms always show expression of β-catenin, thus positive nuclear and cytoplasmic staining for β-catenin are now considered essential diagnostic criteria [2]. Immunoreactivity for cytokeratins, synaptophysin, and CD56 can be observed in 30% to 70% of cases, whereas chromogranin is usually negative [2]. The tumour cells also express vimentin, CD10, CD99, CD56, alpha1-antitrypsin, and progesterone receptors [2]. In the present review, β-catenin was always positive when performed, whereas progesterone receptors were detected in 77.8% of cases. Tien et al. [38] found no difference in immunostaining for sex hormone-receptor proteins, or in pathological features when stratified for gender.

In the present review, 206 (97.2%) patients underwent surgery, which consisted in standard pancreatic resections in 90.4% of cases. Tumour enucleation and incomplete excision should be avoided, due to the risk of tumour dissemination and a high recurrence rate [18]. Some authors proposed extended and more radical surgery in men with SPNs, due to the high likelihood of aggressive disease [15], but no significant differences in terms of follow-up outcomes have been demonstrated between male and female patients [39]. Surgery continues to be considered the standard of care for localised SPNs, and it is accepted for metastatic disease [40]. In the present review, among 10 patients who presented with distant (mostly liver) metastases, four patients underwent surgery of the primary SPN, and two of them were still alive with disease 29 and 70 months after surgery, respectively.

No established chemotherapy regimen is currently recommended for SPNs, but in case of metastatic disease, combined systemic and loco-regional treatments may be used with a palliative intent. Among 10 patients presenting with distant metastases, seven received a combined treatment (i.e. resective or palliative surgery, chemotherapy, and/or TAE), and three of them died of disease after a mean follow-up of 12 months. Concerning 14 patients with disease recurrence (mean DFS 43.1 months), six received a combined treatment (i.e. chemotherapy, TAE, radiotherapy, and/or re-do surgery), and five of them were alive with disease up to 180 months after surgery. Aggressive SPNs (defined as SPNs that locally invaded, recurred, or metastasised) showed a 5- and 10-year survival rate of 71.1% and 65.5%, respectively [41].

To date, a standardised follow-up protocol is not available, but a long follow-up should be performed as SPN recurrence may occur even 10 years after resection [42]. In our review, 14 (7.2%) patients showed a disease recurrence (i.e. liver, or peritoneum), after a mean DFS of 43.1 months (up to 96 months) after surgery. The vast majority (89.5%) of patients were alive with no evidence of disease after a mean follow-up of 47 (range 4–180) months. This finding confirms that SPNs may have an excellent prognosis after radical surgery [18].

Limitations of this systematic review include the variation in the extent and quality of variables collected. Moreover, many institutional series presenting aggregate data of female and male patients had to be excluded from the present review.

In conclusion, pancreatic SPN in males occur mainly (73.8%) in adult patients rather than in childhood. In case of detection of a solid-cystic pancreatic mass, a diagnosis of SPN should be taken in account even in asymptomatic males with a large tumour size. Cytopathologic examination may help the therapeutic planning, particularly in case of metastatic or non-resectable disease. A high hypermetabolism at ^18^F-FDG PET (CT or MRI) strongly suggests a SPN in the differential diagnosis with a pancreatic NEN. Surgery is the treatment of choice in pancreatic SPN, and a long disease-free survival up to 180 months may be achieved after radical resection. In metastatic setting, a multimodal treatment may provide a long-term survival up to 70 months.

## Electronic supplementary material

Below is the link to the electronic supplementary material.

Supplementary file1 (DOCX 17 kb)

Supplementary file2 (DOCX 57 kb)
