# Supplementary material for: Pancreatic solid pseudopapillary neoplasm in male patients: systematic review with three new cases
Source: Updates Surg. 2020 Oct 29;73(4):1285–95. doi: 10.1007/s13304-020-00905-4 (PMC8397648; doi:10.1007/s13304-020-00905-4)
Supplement: Supplementary file 1 — Supplementary file1 (DOCX 17 kb) [file 13304_2020_905_MOESM1_ESM.docx]

**Appendix 1**

SEARCH STRATEGY

**PubMed: (Retrieved 1334 articles)**

(pancreas OR pancreatic) AND (“solid pseudopapillary” OR pseudopapillary OR “pseudo-papillary” OR “solid cystic” OR “solid-cystic” OR “papillary cystic” OR “papillary-cystic”)

Filters applied: English Language. Custom date range: From 01/01/1980 to 05/12/2020.

**SCOPUS: (Retrieved 1811 articles)**

( TITLE-ABS-KEY ( ( {pancreas}  OR  {pancreatic} ) )  AND  TITLE-ABS-KEY ( ( {solid pseudopapillary}  OR  {pseudopapillary}  OR  {pseudo-papillary}  OR  {solid cystic}  OR  {solid and cystic}  OR  {solid-cystic}  OR  {cystic and solid}  OR  {papillary cystic}  OR  {papillary and cystic}  OR  {papillary-cystic}  OR  {solid and papillary} ) ) )

AND  DOCTYPE ( ar  OR  re )  AND  PUBYEAR  >  1979  AND  ( LIMIT-TO ( LANGUAGE ,  "English" ) )
