# Supplementary material for: Pancreatic solid pseudopapillary neoplasm in male patients: systematic review with three new cases
Source: Updates Surg. 2020 Oct 29;73(4):1285–95. doi: 10.1007/s13304-020-00905-4 (PMC8397648; doi:10.1007/s13304-020-00905-4)
Supplement: Supplementary file 2 — Supplementary file2 (DOCX 57 kb) [file 13304_2020_905_MOESM2_ESM.docx]

**APPENDIX 2**

LIST OF ARTICLES INCLUDED IN THE REVIEW

|  | **Publication** | **Males / Total** | **Symptoms reported** | **Imaging reported** | **Surgery reported** | **Immunohistochemistry reported** | **Follow-up reported** |
| --- | --- | --- | --- | --- | --- | --- | --- |
| 1 | Matsunou H, et al. Solid, infiltrating variety of papillary cystic neoplasm of the pancreas. Cancer. 1990;65:2747-57 | 2 / 4 | yes | yes | yes | yes | yes |
| 2 | Cohen RC, et al. S. [Frantz's tumour in a boy - Case report with a review of the paediatric literature](https://www.scopus.com/record/display.uri?eid=2-s2.0-0025843503&origin=resultslist). Pediatric Surgery International | 1 | yes | yes | yes | yes | yes |
| 3 | Klöppel G, et al. Solid-cystic (papillary-cystic) tumours within and outside the pancreas in men: report of two patients. Virchows Arch A Pathol Anat Histopathol. 1991;418 179-83 | 2 | yes | yes | yes | yes | yes |
| 4 | Tsunoda T, et al. Solid and cystic tumor of the pancreas in an adult male. Acta Pathol Jpn. 1991;41:763-70 | 1 | yes | yes | yes | yes | yes |
| 5 | Pettinato G, et al. Papillary cystic tumor of the pancreas. A clinicopathologic study of 20 cases with cytologic, immunohistochemical, ultrastructural, and flow cytometric observations, and a review of the literature. Am J Clin Pathol. 1992;98:478-88. Erratum in: Am J Clin Pathol 1993;99:764 | 1 / 20 | yes | yes | yes | no | yes |
| 6 | Tomioka T, et al. [Solid and cystic tumor of the pancreas occurring without cyst formation in an adult male](https://www.scopus.com/record/display.uri?eid=2-s2.0-0027423501&origin=resultslist). International Journal of Pancreatology. 1993;14:195-200 | 1 | yes | yes | yes | yes | yes |
| 7 | Zamboni G, et al. Expression of progesterone receptors in solid-cystic tumour of the pancreas: a clinicopathological and immunohistochemical study of ten cases. Virchows Arch A Pathol Anat Histopathol. 1993;423:425-31 | 1 / 10 | yes | yes | yes | yes | yes |
| 8 | Cho NH, et al. Correlation between proliferating index and prognostic factors in papillary cystic tumors of the pancreas. J Korean Med Sci. 1995;10:342-51 | 1 / 15 | no | yes | yes | no | yes |
| 9 | Ohiwa K, et al. Solid and cystic tumor (SCT) of the pancreas in an adult man. HPB Surg. 1997;10:315-21 | 1 | yes | yes | yes | yes | yes |
| 10 | Ky A, et al. Experience with papillary and solid epithelial neoplasms of the pancreas in children. J Pediatr Surg. 1998;33:42-4 | 2 / 10 | yes | yes | yes | yes | yes |
| 11 | Wang KS, et al. Papillary cystic neoplasm of the pancreas: a report of three pediatric cases and literature review. J Pediatr Surg. 1998;33:842-5 | 1 / 3 | yes | yes | yes | yes | no |
| 12 | Ferlan-Marolt V, et al. Solid papillary-cystic tumor of the pancreas. Hepatogastroenterology. 1999;46:2978-82 | 1 / 3 | yes | yes | yes | yes | yes |
| 13 | Herskovits M, et al. Papillary cystic neoplasm of the pancreas in a teenage boy. Eur Radiol. 1999;9:1354-6. | 1 | yes | yes | yes | yes | yes |
| 14 | Jung, et al. [Solid and papillary epithelial neoplasm of the pancreas in children](https://www.scopus.com/record/display.uri?eid=2-s2.0-0033062253&origin=resultslist). World Journal of Surgery. 1999;23:233-236 | 2 / 6 | yes | yes | yes | no | yes |
| 15 | Takahashi H, et al. Solid cystic tumor of the pancreas in elderly men: report of a case. Surg Today. 1999;29:1264-7 | 1 | yes | yes | yes | yes | yes |
| 16 | Acebo E, et al. Papillary cystic tumor of the pancreas coexisting with hairy cell leukemia. Pathology. 2000;32:216-9 | 1 | yes | yes | yes | yes | yes |
| 17 | Shorter NA, et al. Malignant pancreatic tumors in childhood and adolescence: The Memorial Sloan-Kettering experience, 1967 to present. J Pediatr Surg. 2002;37:887-92 | 2 / 7 | yes, 1  no, 1 | yes | yes | no | yes, 1  no, 1 |
| 18 | Uchimi K, et al. Solid cystic tumor of the pancreas: report of six cases and a review of the Japanese literature. J Gastroenterol. 2002;37:972-80 | 1 / 6 | yes | yes | yes | yes | no |
| 19 | Wijewardena HC, et al. Three cases of solid cystic pancreatic neoplasm. Ceylon Med J. 2002;47:139-41 | 1 / 3 | yes | yes | yes | no | yes |
| 20 | Ng KH, et al. Solid pseudopapillary tumour of the pancreas. ANZ J Surg. 2003;73:410-5 | 1 / 6 | yes | yes | yes | yes | yes |
| 21 | Potrc S, et al. Urgent Whipple resection for solid pseudopapillary tumor of the pancreas. J Hepatobiliary Pancreat Surg. 2003;10:386-9 | 1 | yes | yes | yes | yes | yes |
| 22 | Chen C, et al. Melanocytic differentiation in a solid pseudopapillary tumor of the pancreas. J Gastroenterol. 2004;39:579-83 | 1 | yes | yes | yes | yes | yes |
| 23 | Mancini GJ, et al. Solid-pseudopapillary tumor of the pancreas: two cases in male patients. Am Surg. 2004;70:29-31 | 2 | yes | yes | yes | yes | yes |
| 24 | Meshikhes AW, Atassi R. Pancreatic pseudopapillary tumor in a male child. JOP. 2004;5:505-11 | 1 | yes | yes | yes | no | yes |
| 25 | Zeytunlu M, et al. [Solid and cystic papillary neoplasms of the pancreas: Report of four cases](https://www.scopus.com/record/display.uri?eid=2-s2.0-33845220734&origin=resultslist). Turkish Journal of Gastroenterology. 2004;15:178-82 | 1 / 4 | yes | yes | yes | yes | yes |
| 26 | Alexandrescu DT, et al. Metastatic solid-pseudopapillary tumour of the pancreas: clinico-biological correlates and management. Clin Oncol (R Coll Radiol). 2005;17:358-63 | 1 / 2 | yes | yes | yes | yes | yes |
| 27 | Huang HL, et al. Solid-pseudopapillary tumor of the pancreas: clinical experience and literature review. World J Gastroenterol. 2005;11:1403-9 | 1 / 7 | yes | yes | yes | yes | yes |
| 28 | Albores-Saavedra J, et al. The clear cell variant of solid pseudopapillary tumor of the pancreas: a previously unrecognized pancreatic neoplasm. Am J Surg Pathol. 2006;30:1237-42 | 1 / 3 | yes | yes | yes | yes | no |
| 29 | Aydiner F, et al. Solid pseudopapillary tumor of the pancreas: emphasis on differential diagnosis from aggressive tumors of the pancreas. Turk J Gastroenterol. 2006;17:219-22 | 1 / 3 | yes | yes | yes | yes | yes |
| 30 | Catalano G, et al. Solid papillary neoplasm of the pancreas: a case report. Tumori. 2006;92:459-61 | 1 | yes | yes | yes | yes | yes |
| 31 | Shabbir A, et al. [Review of solid-pseudopapillary tumour of the pancreas with case illustration](https://www.scopus.com/record/display.uri?eid=2-s2.0-33749036834&origin=resultslist). Surgical Practice. 2006;10:148-53 | 1 | yes | yes | yes | yes | yes |
| 32 | Takahashi Y, et al. Solid-pseudopapillary neoplasms of the pancreas in men and women: do they differ? Virchows Arch. 2006;448:561-9 | 7 / 14 | no | yes | yes | yes | yes |
| 33 | Vargas-Serrano B, et al. Four cases of solid pseudopapillary tumors of pancreas: imaging findings and pathological correlations. Eur J Radiol. 2006;58:132-9 | 1 / 4 | yes | yes | yes | yes | yes |
| 34 | Hu JC, et al. A case of solid pseudopapillary tumour of the pancreas and malignant mesothelioma. J Gastrointest Cancer. 2007;38:71-3 | 1 | yes | yes | yes | yes | yes |
| 35 | Rutland B, et al. Solid-pseudopapillary tumor: a report of three cases in adult males diagnosed utilizing three different modalities. Diagn Cytopathol. 2007;35:234-8 | 3 | yes | Yes, 2  No, 1 | Yes, 2  No, 1 | yes | Yes, 1  No, 2 |
| 36 | Shimizu T, et al. Clinical potential of an antitumor drug sensitivity test and diffusion-weighted MRI in a patient with a recurrent solid pseudopapillary tumor of the pancreas. J Gastroenterol. 2007;42:918-22 | 1 | no | yes | yes | no | yes |
| 37 | Kang CM, et al. A case of spleen-preserving laparoscopic distal pancreatectomy and concomitant cholecystectomy in male patient with solid pseudopapillary neoplasm of the pancreas and gallstone. J Laparoendosc Adv Surg Tech A. 2008;18:259-65 | 1 | yes | yes | yes | no | yes |
| 38 | Fais PO, et al. Is laparoscopic management suitable for solid pseudo-papillary tumors of the pancreas? Pediatr Surg Int. 2009;25:617-21 | 1 / 3 | yes | yes | yes | no | yes |
| 39 | Snajdauf J, et al. Duodenum-sparing technique of head resection in solid pseudopapillary tumor of the pancreas in children. Eur J Pediatr Surg. 2009;19:354-7 | 1 / 13 | yes | yes | yes | no | yes |
| 40 | Yu DC, et al. Childhood pancreatic tumors: a single institution experience. J Pediatr Surg. 2009;44:2267-72 | 1 / 4 | yes | yes | yes | no | yes |
| 41 | A-Cienfuegos J, et al. Solid pseudopapillary tumor of the pancreas (SPPT). Still an unsolved enigma. Rev Esp Enferm Dig. 2010;102:722-8 | 1 / 3 | yes | yes | yes | no | yes |
| 42 | Lin MY, Stabile BE. Solid pseudopapillary neoplasm of the pancreas: a rare and atypically aggressive disease among male patients. Am Surg. 2010;76:1075-8 | 2 / 11 | no | yes | yes | no | yes |
| 43 | Matsuda I, et al. Solid-pseudopapillary neoplasm of the pancreas with massive central calcification in an old man. Pathol Res Pract. 2010;206:372-5 | 1 | yes | yes | yes | yes | yes |
| 44 | Ansari D, et al. Single-institution experience with solid pseudopapillary neoplasm of the pancreas. Scand J Gastroenterol. 2011;46:1492-7 | 3 / 16 | no | yes | yes | no | yes |
| 45 | Imamura N, et al. Synchronous solid pseudopapillary neoplasm and intraductal papillary mucinous neoplasm of the pancreas: report of a case. Surg Today. 2011;41:865-71 | 1 | yes | yes | yes | yes | yes |
| 46 | Ishikawa T, et al. A case of solid-pseudopapillary neoplasm, focusing on contrast-enhanced endoscopic ultrasonography. J Med Ultrason (2001). 2011;38:209 | 1 | yes | yes | yes | yes | no |
| 47 | Marchegiani G, et al. Surgical treatment of pancreatic tumors in childhood and adolescence: uncommon neoplasms with favorable outcome. Pancreatology. 2011;11:383-9 | 2 / 12 | yes | yes | yes | no | yes |
| 48 | Munding J, et al. Solid pseudopapillary tumors of the pancreas: a case series, comparison of histopathological and clinical data. Z Gastroenterol. 2011;49:1417-22 | 1 / 8 | yes | yes | yes | yes | yes |
| 49 | Zganjer M, et al. [Frantz Tumor - pseudopapillary cystic neoplasm of the pancreas in children: Report of two patients](https://www.scopus.com/record/display.uri?eid=2-s2.0-79960799392&origin=resultslist). Acta Medica Saliniana. 2011;40:35-38 | 1 / 2 | yes | yes | yes | no | yes |
| 50 | Bouassida M, et al. Solid pseudopapillary neoplasm of the pancreas in an old man: age does not matter. Pan Afr Med J. 2012;13:8 | 1 | yes | yes | yes | yes | yes |
| 51 | Muller CO, et al. Pancreatic resections for solid or cystic pancreatic masses in children. J Pediatr Gastroenterol Nutr. 2012;54:369-73 | 2 / 7 | yes | yes | yes | no | yes |
| 52 | Tanino M, et al. A case of clear cell variant of solid-pseudopapillary tumor of the pancreas in an adult male patient. Ann Diagn Pathol. 2012;16:134-40 | 1 | yes | yes | yes | yes | no |
| 53 | Ye J, et al. Solid-pseudopapillary tumor of the pancreas: clinical features, pathological characteristics, and origin. J Surg Oncol. 2012;106:728-35 | 12 / 70 | no | yes | yes | no | yes |
| 54 | Yoon HJ, Lim JH. Solid pseudopapillary tumor of the pancreas with hepatic metastasis: spontaneous regression over 10-year follow-up period. Korean J Radiol. 2012;13:648-51 | 1 | yes | yes | yes | no | yes |
| 55 | Attaallah W, et al. Locally advanced pseudopapillary neoplasm of the pancreas in a male patient: a case report. JOP. 2013;14:438-41 | 1 | yes | yes | yes | yes | yes |
| 56 | Hirabayashi K, et al. Synchronous pancreatic solid pseudopapillary neoplasm and intraductal papillary mucinous neoplasm. World J Gastroenterol. 2013;19:3358-63 | 1 | yes | yes | yes | yes | yes |
| 57 | Laje P, et al. Solid pseudopapillary neoplasm of the pancreas in children: a 15-year experience and the identification of a unique immunohistochemical marker. J Pediatr Surg. 2013;48:2054-60 | 1 / 6 | yes | yes | yes | yes | yes |
| 58 | Li HX, et al. Multi-centric solid-pseudopapillary neoplasm of the pancreas. Med Oncol. 2013;30:330 | 1 / 7 | yes | yes | yes | yes | yes |
| 59 | Maimone A, et al. Preoperative diagnosis of a solid pseudopapillary tumour of the pancreas by Endoscopic Ultrasound Fine Needle Biopsy: A retrospective case series. Dig Liver Dis. 2013;45:957-60 | 1 / 5 | yes | yes | yes | yes | yes |
| 60 | Nasit JG, et al. Solid pseudopapillary tumor of pancreas in a male child: a diagnosis by fine needle aspiration cytology. Fetal Pediatr Pathol. 2013;32:265-70 | 1 | yes | yes | yes | yes | yes |
| 61 | Park HY, et al. Endoscopic ultrasound-guided fine needle aspiration of solid pseudopapillary tumors of the pancreas: a report of three cases. Korean J Intern Med. 2013;28:599-604 | 1 / 3 | yes | yes | yes | yes | no |
| 62 | Rodrigues-Duarte H, et al. Solid pseudopapillary tumor of the pancreas in a child: imaging findings with diffusion-weighted MR imaging. JOP. 2013;14:195-8 | 1 | yes | yes | yes | no | no |
| 63 | Saigo C, et al. Two rare cases of a solid pseudopapillary neoplasm of the pancreas. Oncol Lett. 2013;6:871-874 | 1 / 2 | yes | yes | yes | yes | yes |
| 64 | Yagcı A, et al. Diagnosis and treatment of solid pseudopapillary tumor of the pancreas: experience of one single institution from Turkey. World J Surg Oncol. 2013;11:308 | 1 / 10 | no | yes | yes | yes | yes |
| 65 | Cai YQ, et al. Solid pseudopapillary tumor of the pancreas in male patients: report of 16 cases. World J Gastroenterol. 2014;20:6939-45 | 16 / 116 | yes | yes | yes | yes | yes |
| 66 | Chen XM, et al. Laparoscopic central pancreatectomy for solid pseudopapillary tumors of the pancreas: our experience with ten cases. World J Surg Oncol. 2014;12:312 | 2 / 10 | no | yes | yes | no | yes |
| 67 | Estrella JS, et al. Solid pseudopapillary neoplasm of the pancreas: clinicopathologic and survival analyses of 64 cases from a single institution. Am J Surg Pathol. 2014;38:147-57 | 10 / 64 | Yes, 6  No, 4 | Yes, 9  No, 1 | yes | yes | Yes, 9  no, 1 |
| 68 | Fujii M, et al. A solid pseudopapillary neoplasm without cysts that occurred in a patient diagnosed by endoscopic ultrasound-guided fine-needle aspiration: a case report. J Med Case Rep. 2014;8:243 | 1 | yes | yes | yes | yes | yes |
| 69 | Garg B, et al. Solid pseudopapillary tumour of pancreas: a report of 5 cases. J Assoc Physicians India. 2014;62:61-4 | 1 / 5 | yes | yes | yes | no | yes |
| 70 | Hosokawa I, et al. Preoperative diagnosis and surgical management for solid pseudopapillary neoplasm of the pancreas. J Hepatobiliary Pancreat Sci. 2014;21:573-8 | 4 / 10 | yes | yes | yes | yes | yes |
| 71 | Liu QY, et al. Computed tomography and magnetic resonance imaging findings of malignant solid pseudopapillary tumors of the pancreas with macroscopic venous tumor thrombosis: a report of 4 cases. J Comput Assist Tomogr. 2014;38:383-90 | 1 / 4 | yes | yes | yes | no | yes |
| 72 | Park JY, et al. Solid pseudopapillary tumor of the pancreas in children: 15-year experience at a single institution with assays using an immunohistochemical panel. Ann Surg Treat Res. 2014;86:130-5 | 3 / 11 | yes | yes | yes | yes | yes |
| 73 | Serrano PE, et al. Risk factors associated with recurrence in patients with solid pseudopapillary tumors of the pancreas. JOP. 2014;15:561-8 | 6 / 32 | no | yes | yes | yes | yes |
| 74 | Šileikis A, et al. Solid Pseudopapillary Neoplasm of the Pancreas: Analysis of Seven Cases. Viszeralmedizin. 2014;30:211-5 | 1 / 7 | no | yes | yes | yes | no |
| 75 | Suzuki S, et al. Clinical and pathological features of solid pseudopapillary neoplasms of the pancreas at a single institution. Dig Surg. 2014;31:143-50 | 5 / 34 | no | yes | yes | yes | yes |
| 76 | Tsukamoto M, et al. Clinical features and management of pancreatic solid pseudopapillary tumor. Am Surg. 2014;80:1212-5 | 2 / 6 | yes | yes | yes | yes | yes |
| 77 | Alves JR, Amico EC. [Solid-pseudopapillary neoplasm of the pancreas: Case series and literature review](https://www.scopus.com/record/display.uri?eid=2-s2.0-84929577626&origin=resultslist). Journal of the Pancreas. 2015;16:218-226 | 1 / 10 | no | yes | yes | no | no |
| 78 | Cai Y, et al. Laparoscopic distal pancreatectomy for solid-pseudopapillary tumor of the pancreas. Surg Laparosc Endosc Percutan Tech. 2015;25:e8-e10 | 1 / 4 | yes | yes | yes | no | yes |
| 79 | Inoue T, et al. Solid pseudopapillary neoplasm of the pancreas associated with familial adenomatous polyposis. Intern Med. 2015;54:1349-55 | 1 | yes | yes | yes | yes | no |
| 80 | Ozguven BY, et al. Solid-pseudopapillary neoplasm of the pancreas: Clinicopathologic and immunohistochemical analysis of nine cases. Indian J Pathol Microbiol. 2015;58:292-5 | 1 / 9 | no | yes | yes | yes | Yes |
| 81 | Violari EG, et al. Successful control of liver metastases from pancreatic solid-pseudopapillary neoplasm (SPN) using hepatic arterial embolization. Cardiovasc Intervent Radiol. 2015;38:479-83 | 1 | yes | yes | yes | no | yes |
| 82 | Yan SX, et al. Solid pseudopapillary neoplasm collides with a well-differentiated pancreatic endocrine neoplasm in an adult man: case report and review of histogenesis. Am J Clin Pathol. 2015;143:283-7 | 1 | yes | yes | yes | yes | no |
| 83 | Carlotto JR, et al. Solid pseudopapillary neoplasm of the pancreas. Arq Bras Cir Dig. 2016;29:93-6 | 1 / 17 | yes | yes | yes | no | yes |
| 84 | Ersen A, et al. Solid-Pseudopapillary neoplasm of the pancreas: A clinicopathological review of 20 cases including rare examples. Pathol Res Pract. 2016;212:1052-1058 | 3 / 20 | no | yes | yes | yes | yes |
| 85 | Goh BK, et al. First experience with robotic spleen-saving, vessel-preserving distal pancreatectomy in Singapore: a report of three consecutive cases. Singapore Med J. 2016;57:464-9 | 1 | yes | yes | yes | no | no |
| 86 | Hongjian X, et al. Solid Pseudopapillary Tumor of the Pancreas in a 50-Year-Old Man: A Case Report and Review of the Literature. Case Rep Pancreat Cancer. 2016;2:23-27 | 1 | yes | yes | yes | yes | yes |
| 87 | Kanchana WG, et al. Laparoscopic Resection of Pancreatic Tail Solid Pseudopapillary Tumour in a Young Male. Case Rep Gastrointest Med. 2016;2016:4037618 | 1 | yes | yes | yes | no | no |
| 88 | Lam MC, et al. [Liver metastasis ten years after excision of a solid pseudopapillary neoplasm of the pancreas](https://www.scopus.com/record/display.uri?eid=2-s2.0-84978209317&origin=resultslist). Hong Kong Journal of Radiology. 2016;19:e14-e17 | 1 | yes | yes | yes | no | yes |
| 89 | Mirminachi B, et al. Solid Pseudopapillary Neoplasm of Pancreas; A Case Series and Review Literature. Middle East J Dig Dis. 2016;8:102-8. | 1 / 7 | yes | yes | yes | yes | no |
| 90 | Yang F, et al. Prognostic value of Ki-67 in solid pseudopapillary tumor of the pancreas: Huashan experience and systematic review of the literature. Surgery. 2016;159:1023-31 | 13 / 71 | yes, 5  no, 8 | yes | no | yes | yes |
| 91 | Zhang H, et al. [Clinical-pathological features and therapeutic strategies of malignant solid pseudopapillary tumor of the pancreas: Cases report.](https://www.scopus.com/record/display.uri?eid=2-s2.0-84994320294&origin=resultslist) Int J Clin Exp Med. 2016;9:19826-30 | 1 / 2 | yes | yes | yes | no | yes |
| 92 | Aso A, et al. Solid Pseudopapillary Neoplasm of the Pancreas in Young Male Patients: Three Case Reports. Case Rep Gastrointest Med. 2017;2017:9071678 | 3 | yes | yes | no | yes | yes |
| 93 | Bao L, et al. [Solid pseudopapillary neoplasm of the pancreas in children: A clinical and pathological study of 16 cases](https://www.scopus.com/record/display.uri?eid=2-s2.0-85016956095&origin=resultslist). Int J Clin Exp Pathol. 2017;10:3199-3208 | 5 / 16 | yes | yes | yes | yes | yes |
| 94 | Bhutani N, et al. Solid pseudopapillary tumor of the pancreas: Experience at a tertiary care centre of Northern India. Int J Surg Case Rep. 2017;39:225-230 | 1 / 11 | no | yes | yes | yes | no |
| 95 | Chikuie E, et al. A solid pseudopapillary neoplasm of the pancreas in a man presenting with acute pancreatitis: A case report. Int J Surg Case Rep. 2017;31:114-8 | 1 | yes | yes | yes | yes | no |
| 96 | Limaiem F, et al. Solid pseudopapillary neoplasm of the pancreas in two male patients: gender does not matter. Pan Afr Med J. 2017;27:283 | 2 | yes | yes | yes | yes | no |
| 97 | Uçar A, et al. Synchronous Solid Pseudopapillary Tumor and Insulinoma in an Adolescent MEN1 Patient Presenting with Diagnostic Dilemmas. J Clin Res Pediatr Endocrinol. 2017;9:375-379 | 1 | yes | yes | yes | yes | yes |
| 98 | Watanabe Y, et al. A case of aggressive solid pseudopapillary neoplasm: Comparison of clinical and pathologic features with non-aggressive cases. Pathol Int. 2017;67:202-7 | 7 / 11 | no | yes | yes | yes | yes |
| 99 | Yao L, et al. Radical resection and enucleation in Chinese adolescents with pancreatic tumors: A 15-year case series. Medicine (Baltimore). 2017;96):e6438 | 1 / 5 | yes | yes | yes | no | yes |
| 100 | Cuccurullo D, et al. Solid pancreatic pseudopapillary tumor managed laparoscopically: A case report and review of the literature. Int J Surg Case Rep. 2018;45:4-8 | 1 | yes | yes | yes | yes | no |
| 101 | Goumard C, et al. Tips and tricks of splenic vessel preservation during laparoscopic distal pancreatectomy. Surg Endosc. 2018;32:2149-2150 | 1 | yes | yes | yes | no | no |
| 102 | Hao EIU, et al. Aggressiveness of solid pseudopapillary neoplasm of the pancreas: A literature review and meta-analysis. Medicine (Baltimore). 2018;97:e13147 | 2 / 8 | no | yes | yes | no | yes |
| 103 | Kayar Y, et al. [Evaluation of solid pseudopapillary tumor of the pancreas: One tertiary center experience](https://www.scopus.com/record/display.uri?eid=2-s2.0-85047162943&origin=resultslist). Biomedical Research (India). 2018;29:1298-1303 | 1 / 5 | yes | yes | yes | yes | yes |
| 104 | McCluney S, et al. Solid pseudopapillary tumour of the pancreas: clinicopathological analysis. ANZ J Surg. 2018;88:891-895 | 1 / 11 | yes | yes | yes | no | yes |
| 105 | Ozcan A, et al. Evaluation of childhood solid pseudopapillary tumors of the pancreas. North Clin Istanb. 2018;5:207-10 | 2 / 6 | yes | yes | yes | yes | yes |
| 106 | Portelli M, et al. [Pseudopapillary tumours of the pancreas: A case series and short review of the literature](https://www.scopus.com/record/display.uri?eid=2-s2.0-85047164577&origin=resultslist). European Surgery - Acta Chirurgica Austriaca. 2018;50:189-94 | 1 / 3 | yes | yes | yes | yes | yes |
| 107 | Scandavini C, et al. Pancreatectomies for pancreatic neoplasms in pediatric and adolescent age: A single institution experience. Pancreatology. 2018;18:204-207 | 2 / 9 | no | yes | yes | no | yes |
| 108 | Sharma PK, et al. Recurrent Solid Pseudopapillary Neoplasm of Pancreas: Case Report and Review of Literature. J Pancreat Cancer. 2018;4:25-29 | 1 | yes | yes | yes | yes | yes |
| 109 | Varshney A, et al. Whipple's Pancreaticoduodenectomy in Pediatric Patients: An Experience from a Tertiary Care Center. J Indian Assoc Pediatr Surg. 2018;23:212-15 | 1 | yes | yes | yes | no | yes |
| 110 | Campos M, et al. Tackling the Diagnosis: Solid Pseudopapillary Tumor of the Pancreas in a Young Man. Gastroenterology Res. 2019;12:174-5 | 1 | yes | yes | yes | yes | no |
| 111 | Chon HK, et al. An Unusual Presentation of a Solid Pseudopapillary Tumor of the Pancreas Mimicking Adenocarcinoma. Clin Endosc. 2019 Nov 22. Epub ahead of print | 1 | yes | yes | yes | yes | yes |
| 112 | Hansen CP, et al. Solid pseudopapillary neoplasm of the pancreas: Clinical-pathological features and management, a single-center experience. Rare Tumors. 2019;11:2036361319878513 | 3 / 15 | no | yes | yes | yes | yes |
| 113 | Kovler ML, et al. Laparoscopic Spleen-Preserving Distal Pancreatectomy for Solid Pseudopapillary Neoplasm in Adolescents. J Laparoendosc Adv Surg Tech A. 2019;29:1372-7 | 2 / 3 | yes | yes | yes | no | yes |
| 114 | Policarpio-Nicolas MLC, et al. Pleomorphic and atypical multinucleated giant cells in solid pseudopapillary neoplasm of pancreas: A diagnostic pitfall in cytology and a review of the literature. Diagn Cytopathol. 2019;47:488-93 | 1 | yes | no | no | yes | no |
| 115 | Torres OJM, et al. Pancreatoduodenectomy for solid pseudopapillary tumor of the pancreas: a multi-institution study. Arq Bras Cir Dig. 2019;32:e1442 | 1 / 16 | yes | yes | yes | no | yes |
| 116 | Yalçın B, et al. Solid pseudopapillary neoplasm of the pancreas in children: Hacettepe experience. ANZ J Surg. 2019;89:E236-E240 | 3 / 19 | yes | yes | yes | no | yes |
| 117 | Yang HB, et al. Pediatric minimally invasive surgery for malignant abdominal tumor: Single center experience. Medicine (Baltimore). 2019;98:e16776 | 1 / 3 | no | yes | yes | no | yes |
| 118 | Farhat W, et al. Solid pseudopapillary neoplasm of the pancreas: a report of 10 cases and literature review. ANZ J Surg. 2020 Jan 28. Epub ahead of print | 2 / 10 | yes | yes | yes | yes | yes |
| 119 | Guo T, et al. Diagnosis and Surgical Treatment and Pathological Findings of Solid Pseudopapillary Tumor of the Pancreas: A Single-Institution Experience. Cancer Manag Res. 2020;12:581-8 | 16 / 87 | yes | yes | no | no | no |
| 120 | Ihara T, et al. Early Diagnosis of a Pediatric Pancreatic Tumor by Abdominal Physical Examination: A Case Report. J Emerg Med. 2020;58:e211-e214 | 1 | yes | yes | yes | no | yes |
| 121 | Pandit N, et al. Solid Pseudopapillary Neoplasm of the Pancreas: A Distinct Case Series from Eastern Nepal. J Gastrointest Cancer. 2020;51:649-653 | 1 / 4 | yes | yes | yes | no | yes |
| 122 | Yamaguchi H, et al. Seeding of a Tumor in the Gastric Wall after Endoscopic Ultrasound-guided Fine-needle Aspiration of Solid Pseudopapillary Neoplasm of the Pancreas. Intern Med. 2020;59:779-82 | 1 | no | yes | yes | no | yes |
